# Unravelling the adaptation responses to osmotic and temperature stress in *Chromohalobacter salexigens*, a bacterium with broad salinity tolerance

**DOI:** 10.1186/1746-1448-4-14

**Published:** 2008-09-15

**Authors:** Carmen Vargas, Montserrat Argandoña, Mercedes Reina-Bueno, Javier Rodríguez-Moya, Cristina Fernández-Aunión, Joaquín J Nieto

**Affiliations:** 1 Department of Microbiology and Parasitology, Faculty of Pharmacy, University of Seville, 41012 Seville, Spain

## Abstract

*Chromohalobacter salexigens*, a Gammaproteobacterium belonging to the family *Halomonadaceae*, shows a broad salinity range for growth. Osmoprotection is achieved by the accumulation of compatible solutes either by transport (betaine, choline) or synthesis (mainly ectoine and hydroxyectoine). Ectoines can play additional roles as nutrients and, in the case of hydroxyectoine, in thermotolerance. A supplementary solute, trehalose, not present in cells grown at 37°C, is accumulated at higher temperatures, suggesting its involvement in the response to heat stress. Trehalose is also accumulated at 37°C in ectoine-deficient mutants, indicating that ectoines suppress trehalose synthesis in the wild-type strain. The genes for ectoine (*ectABC*) and hydroxyectoine (*ectD*, *ectE*) production are arranged in three different clusters within the *C. salexigens *chromosome. In order to cope with changing environment, *C. salexigens *regulates its cytoplasmic pool of ectoines by a number of mechanisms that we have started to elucidate. This is a highly complex process because (i) hydroxyectoine can be synthesized by other enzymes different to EctD (ii) ectoines can be catabolized to serve as nutrients, (iii) the involvement of several transcriptional regulators (σ^S^, σ^32^, Fur, EctR) and hence different signal transduction pathways, and (iv) the existence of post-trancriptional control mechanisms. In this review we summarize our present knowledge on the physiology and genetics of the processes allowing *C. salexigens *to cope with osmotic stress and high temperature, with emphasis on the transcriptional regulation.

## Background

The availability of water in their habitat is one of the most important parameters affecting the survival and growth of microorganisms. Thus, they must be able to cope and adapt to the frequent fluctuations in the water content of the external environment to maintain their turgor pressure within limits necessary for growth and multiplication. Osmoadaptation mechanisms are referred as those physiological and genetic manifestations of adaptation to low and high water environments [1]. Prokaryotic osmoadaptation has recently gained considerable importance not only to understand how bacterial cells cope with environmental conditions but also because this knowledge can be applied in agriculture, food and fermentation industries, and biomedicine [2].

The intracellular accumulation of large quantities of a particular group of small, organic osmolytes is a common and flexible strategy of osmoadaptation for eukaryotic and prokaryotic microorganisms when they need to cope with hyperosmotic conditions. These compounds, which function as osmoprotectants, are termed compatible solutes since they can be amassed by the cell in very high concentrations providing osmotic balance without disturbing essential cellular functions and the correct folding of proteins [3]. Since the cell may also release these solutes upon a hypoosmotic shock by using specific efflux systems restoring the osmotic balance, this strategy enables organisms to adapt to a wide range of salt concentrations by adjusting the cytoplasmic solute pool to the osmolality of the surrounding environment. For this reason, it is not surprising that this is an evolutionary well-conserved adaptation strategy in a wide diversity of microorganisms [4-6]. Although there is a relatively high diversity of compounds reported as compatible solutes, they are mainly sugars (sucrose and trehalose), polyols (glycerol, glucosylglycerol, mannosylglycerol, and arabitol, among others), amino acids (glutamine and derivatives, proline, alanine), quaternary amines (betaines, choline) and ectoines (ectoine and β-hydroxyectoine). Sometimes they are accumulated after their transport when present in the medium or, in some cases, an external precursor is taken up and later on it is converted into the specific compatible solute. Other compounds can only be accumulated after their synthesis *de novo*, by using a specific biosynthetic pathway, i.e., when cells are growing in a minimal medium. In any case, it is generally assumed that uptake of external osmoprotectants in the medium is energetically preferred over synthesis *de novo *[4,7-9]. Recently there is an increasing body of evidence indicating that compatible solutes are multifunctional molecules with stabilizing properties, hence protecting cell components from several abiotic stresses such as high salinity, freezing, desiccation, high temperature, pressure, or oxygen radicals, functioning as chemical chaperones [10]. This multi-stress protection is especially remarkable in the case of some of them, such as trehalose and ectoines [11,12], which have current and potential applications in molecular biology, agriculture, biotechnology and biomedicine [13,14]. In some circumstances, some of these osmoprotectants can be also used as efficient carbon and nitrogen sources, such as the ectoines or betaines, which are widespread in nature [9,15].

Moderately halophilic bacteria (growing optimally between 0.5 and 2.5 M salt) [16] of the family *Halomonadaceae*, such as *Halomonas elongata *and *Chromohalobacter salexigens *(formerly *H. elongata *DSM 3043) [17], show a remarkable versatility with respect to their salt tolerance and have been extensively used in recent years to study the bacterial osmoadaptation processes [16]. More than a decade ago, we selected *C. salexigens *as a model organism for such studies due to the following reasons. First, it has an unusual extremely broad salinity range, one of the widest found in Nature, growing in the presence of NaCl concentrations ranging from ca. 0.1 to 4 M in complex medium in a temperature range of 15 to 45°C [17], and from 0.5 to 3 M in minimal medium M63, with optimal growth at 1.5 M NaCl and 37°C [18]. It is worth mentioning that *C. salexigens *is a true halophile, requiring at least 0.5 M NaCl for any growth at all in minimal medium [18]. Interestingly, 0.5 M NaCl is the maximal salt concentration that the non halophilic *E. coli*, traditionally used for osmoregulation studies, can tolerate [1]. It has been recently reported that while *C. salexigens *needs moderate concentrations of Na^+ ^and Cl^- ^ions, its growth rate was stimulated by a number of other salts, indicating that it requires a combination of NaCl and high ionic strength for optimal growth [19]. Secondly, *C. salexigens *is a metabolically versatile bacterium, growing fast on a wide range of simple carbon compounds as its sole carbon and energy source [17]. Another advantage is that tools that allow the genetic manipulation of *C. salexigens *have been largely developed [20]. Finally, the *C. salexigens *complete genome sequence has recently been determined by the Joint Genome Institute (an automatically generated annotation is available at http://genome.ornl.gov/microbial/csal/), enabling an *in silico *search for systems involved in halophilic behaviour and halotolerance.

Although adaptive changes in its membrane lipid composition contribute to the long-term response of *C. salexigens *to salt stress [21], osmoadaptation is mainly achieved upon accumulation of compatible solutes, after their *de novo *synthesis or by transport from the surrounding media when available. The main endogenous compatible solutes are ectoines (ectoine and hydroxyectoine) and a mutant unable to synthesize them cannot grow above 0.75 M NaCl in a minimal medium such as M63, in contrast to the wild type strain, which grows well up to 3 M NaCl in the same medium [22]. Here we summarize our present knowledge on the response of *C. salexigens *to high salinity and high temperature. Our data show that the synthesis of ectoines by this extremophile is a highly complex process that is both osmo- and thermoregulated, and controlled at both the transcriptional and post-trancriptional level.

### Uptake of osmoprotectants from environmental sources

**Figure 1:**
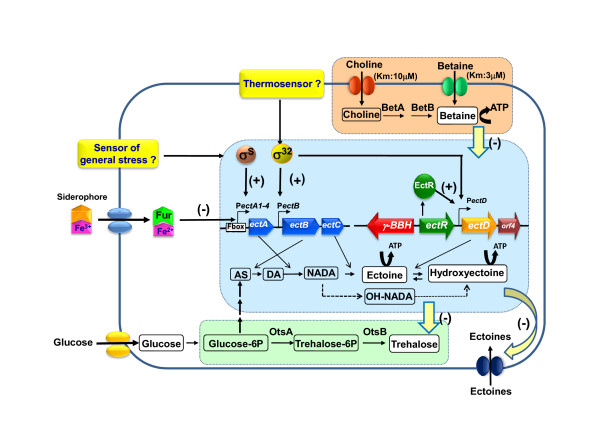
**Hierarchical accumulation of the compatible solutes betaine, ectoine, hydroxyectoine and trehalose by *Chromohalobacter salexigens***. Betaine is accumulated by transport or after choline oxidation. Ectoines and trehalose are synthesized *de novo*. Betaine inhibits ectoine(s) synthesis and ectoine(s) suppress trehalose accumulation. In addition to osmoprotectants, betaine, its precursor choline, and ectoines, can be used as carbon sources (this has been represented by a curved arrows plus ATP close to ectoine, hydroxyectoine and betaine). The central scheme summarizes the present knowledge on the transcriptional mechanisms governing ectoine(s) synthesis. Abbreviations: AS, L-aspartate β-semialdehyde; γ-BBH, γ-butirobetaine hydroxylase; DA, L-2,4-diaminobutyrate; NADA, Nγ-Acetyl-L-2,4-diaminobutyrate; OH-NADA, 3-Hydroxy-Nγ-Acetyl-L-2,4-diaminobutyrate.

*C. salexigens *accumulates externally supplied glycine betaine (named betaine hereafter) either by direct transport or after oxidation of its precursor choline in a two-step enzymatic pathway that has been characterized at the biochemical and molecular level (Fig. 1) [23,24]. *C. salexigens *genome carries several set of genes that may encode ABC-type betaine uptake systems (see below), but none of them has been analysed at the molecular level. Gene identifications are available from the author on request, but are not included as annotation is in progress and so identifications are subject to change At the physiological level, we have characterized an osmoregulated high-affinity transport system for betaine (Km = 3.06 μM, Fig. 1), which can also take up ectoine and proline betaine (although with less affinity than for betaine), but not choline, choline-O-sulfate or proline [18]. Since proline can be used as a carbon source for *C. salexigens*, and choline or choline-O-sulfate exert osmoprotective effects, we predicted that *C. salexigens *should possess additional transport systems for these solutes [18]. Within the *C. salexigens *genome, there are at least five genes encoding putative BCCT-type (Betaine/Carnitine/Choline-Transport) transporters orthologs to the BetT system of *E. coli *[25] (none of them linked to the *betIBA *operon encoding choline oxidation), two genes encoding putative Na^+^/proline symporters, and five clusters of genes encoding ABC-type betaine/proline uptake systems. This high abundance of osmoprotectant transport systems may support the exquisite adaptation of *C. salexigens *to hyperosmotic environments.

The *betIBA *genes, which are responsible for the oxidation of choline into betaine, were isolated and their molecular genetics characterized by our team, in collaboration with Erhard Bremer and co-workers (Fig. 1). Remarkably, BetA is able to oxidize both choline and betaine aldehyde and therefore can mediate both steps in the synthesis of betaine [24].

As described before, if present in the surrounding medium, choline-O-sulfate confers osmoprotection [18], but currently it is unknown if choline-O-sulfate is transformed into betaine (via choline, by means of a choline sulphatase activity) or accumulated *per se *as an osmoprotectant. While the absence, within the *C. salexigens *genome, of an ortholog to known *betC *genes (encoding the choline sulphatase) suggests the second option, this needs experimental confirmation.

Ectoine and hydroxyectoine, the main compatible solutes accumulated by *C. salexigens *in response to salinity [22] can also be taken up from the external medium *via *at least one transport system whose activity is maximal at optimal salinity (1.5 M NaCl), and shows 3- and 1.5-fold lower values at 0.75 and 2.5 M NaCl, respectively [26]. In contrast, accumulation of betaine by transport from the external medium gradually increases in response to salinity [18,26]. At optimal salinity, the rate of ectoine transport is double that of betaine. Very interestingly, the *ectA *strain CHR62, which cannot synthesize ectoines, showed a 6.8-fold increased transport rate if compared with the wild type grown at the same salinity. This result suggests that endogenous ectoine(s) may, directly or indirectly, repress its own transport [26]. *C. salexigens *genome carries orthologs to the *H. elongata teaABC *genes (encoding an osmoregulated TRAP-T-type transport system for ectoines [27], but arranged differently (*teaA *in one strand, followed by *teaBC *in the opposite strand), and also one ortholog to the *Marinococcus halophilus ectM *gene for ectoine transport [28]. The characterization of mutants affected in these genes, currently in progress in our laboratory, will give us a clue of which of them is more important for ectoine(s) transport in *C. salexigens*.

### Catabolism of ectoines

In addition to osmoprotection, *C. salexigens *can use proline, choline, betaine [18], ectoine and hydroxyectoine [26] as the sole carbon source, although glucose is the preferred carbon source at any salinity. In agreement with this, glucose partially represses, but does not totally abolish, ectoine catabolism [26]. Ectoine and hydroxyectoine support growth only at optimal salinity (1.5 M NaCl) but not at low (0.75 M) or high (2.5 M) salt. This finding may be correlated with the lower transport rates found at suboptimal salinities [26] and/or control mechanisms repressing ectoine catabolism at high salt, in favour of ectoine(s) accumulation. Within the *C. salexigens *genome sequence we have found a cluster of 11 genes, all oriented in the same direction, which may encode orthologs to EutB, EutC, EutD, and EutE proteins of *Sinorhizobium meliloti*, involved in ectoine degradation [29]. However, we found no hits against EutA, and the *eutBCDE *genes were organized differently, and apart from the *ehu *ectoine transport system found in *S. meliloti*. Although this finding suggests that *S. meliloti *and *C. salexigens *might use the same catabolic routes for ectoines, this requires experimental evidence.

### Synthesis of ectoines

**Figure 2:**
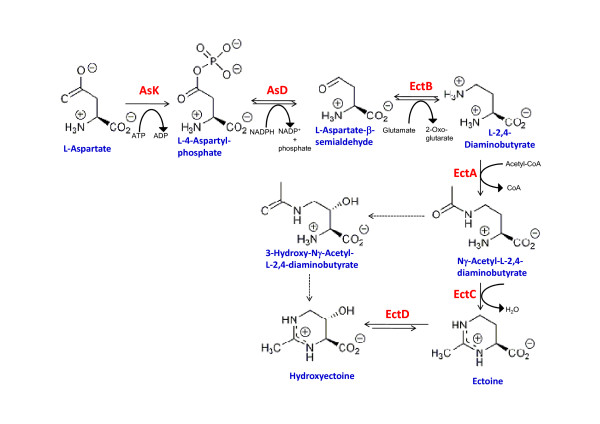
**Biosynthetic pathway for ectoines from aspartate in *C. salexigens***. The solid and dashed lines indicate established and proposed steps, respectively. The enzymes involved are Aspartate kinase (Ask), Aspartate semialdehyde dehydrogenase (Asd), L-diaminobutyric acid transaminase (EctB), L-diaminobutyric acid acetyl transferase (EctA), ectoine synthase (EctC), and ectoine hydroxylase (EctD).

Ectoine and hydroxyectoine are synthesized from aspartate semialdehyde, an intermediate in the biosynthetic route of amino acids derived from aspartic acid (Fig. 2). The biosynthesis of ectoine occurs in three enzymatic steps [30]. First, aspartate semialdehyde is converted into diaminobutyric acid (DA), which is subsequently acetylated to Nγ-acetyldiaminobutyrate (NADA). The cyclic condensation of this compound leads to the formation of ectoine. Hydroxyectoine is synthesized via ectoine hydroxylation, which is a reversible reaction [31]. On the other hand, we have suggested that *C. salexigens *can synthesize hydroxyectoine by an alternative pathway that converts Nγ-acetyldiaminobutyric into hydroxyectoine without the involvement of ectoine [31] (Fig. 2).

**Figure 3:**
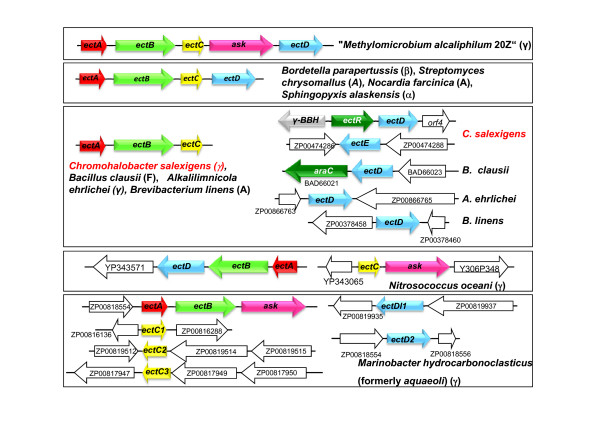
**Genomic context of the genes for ectoine (*ectABC*) and hydroxyectoine (*ectD*) synthesis in a number of ectoine(s) producing bacteria**. Only bacteria whose genome sequence is available are included. The gene *ectE *of *C. salexigens *encodes a second putative ectoine hydroxylase. *asK *encodes the aspartate kinase and *ectR *(in *C. salexigens*) and *araC *(in *B. clausii*) refer to transcriptional regulators of the AraC family. The taxonomic position of the species is abbreviated as follows: A, Actinobacteria, F, Firmicute, α, β,γ, Alpha- Beta-, and Gammaproteobacteria, respectively.

The ectoine synthesis genes (*ectA*, *ectB*, and *ectC*) are very conserved among ectoine-producing bacteria. In *C. salexigens*, they lay within a 2.8-kb region encoding the diaminobutyric acid acetyltransferase (EctA), diaminobutyric acid transaminase (EctB), and ectoine synthase (EctC), respectively [32] (Fig. 3). In addition, we have recently isolated and characterized the *ectD *gene, encoding an ectoine hydroxylase that synthesizes hydroxyectoine from ectoine [33]. *ectD *is preceded by a regulatory gene (preliminarily named *ectR*), which may encode a transcriptional activator of the ectoine hydroxylase gene (Reina-Bueno et al., unpublished results). *C. salexigens *genome contains a second copy *ectD*, which has been named *ectE*, whose expression is negligible if compared to that of *ectD *(Reina-Bueno et al., unpublished results) (Fig. 3). Moreover, our finding that hydroxyectoine accumulation is drastically reduced in the *ectD *insertion mutants strongly indicates that *ectD *is principally responsible for hydroxyectoine synthesis in *C. salexigens *[33]. Whether or not the remaining hydroxyectoine comes from the activity of EctE or from the proposed alternative route is currently under investigation in our laboratory.

The genetic regions involved in ectoine (*ectABC*) and hydroxyectoine (*ectD*, *ectE*) synthesis in *C. salexigens *lay apart within the *C. salexigens *genome. This organization differs from that found in the sequenced genomes of other ectoines-producing bacteria (all belonging to the Firmicutes, Actinobacteria and Proteobacteria), where different genomic contexts can be found. These include from a complete cluster carrying the *ectABC-asK-ectD *genes (*asK *encoding the aspartate kinase) of "*Methylomicrobium alcaliphilum *20Z" (maybe the closest to the ancestral organization) to the set *ectABCD *of some Actinobacteria and Proteobacteria, or the scattering of the genes within the chromosome, with duplications of *ectC *and *ectD *(Fig. 3). Thus, the general assumption that the organization of the ectoine synthesis genes is very well conserved (*ectABC*) should be taken with caution, as when more genomes are sequenced, more variations in this genetic organization may be found.

### Response of *C. salexigens *to hypersaline stress

At the optimal growth temperature (37°C) and in absence of any osmoprotectant, ectoine, followed by hydroxyectoine, and glutamate and glutamine, are the main compatible solutes accumulated by *C. salexigens *at any salinity (from 0.5 to 3 M NaCl) in M63 minimal medium, as shown by ^13^C-NMR analysis [22,32,33]. Low levels of alanine, trehalose and lactate can also accumulate as minor solutes in these conditions. When ectoines are quantified by HPLC, their accumulation is maximal during stationary phase of growth. Higher levels of ectoine are found at any salinity tested, and there is an evident increase of the content of both compatible solutes with increasing salt concentration, with 2.75- and 13.8-fold higher levels of ectoine and hydroxyectoine, respectively, in cells grown at 3 M NaCl, if compared to cells grown at 0.75 M NaCl [33]. Ectoine production is growth-phase dependent in the chloride-dependent Gram-positive halophilic model organism *Halobacillus halophilus*, which produces ectoine predominantly at very high salinities, along with proline [34].

### Response of *C. salexigens *to temperature stress

The ^13^C-NMR spectrum of extracts of *C. salexigens *grown at 45°C with 2.5 M NaCl (the optimal salinity for growth at this temperature) shows the presence of (in this order) hydroxyectoine, ectoine, trehalose, glutamate, and the ectoine precursor, Nγ-acetyldiaminobutiric acid [33]. By using HPLC techniques, we quantified ectoine and hydroxyectoine accumulation. Ectoine was the predominant solute from 20 to 35°C, while the hydroxyectoine levels remained very low at these temperatures. However, the content of ectoine decreased and that of hydroxyectoine increased from 35 to 45°C, resulting in higher levels of hydroxyectoine than that of ectoine in cells grown at 45°C, although the pool of ectoines remained constant at a given salinity. These data indicate that the accumulation of hydroxyectoine in *C. salexigens *is up-regulated by temperature [33]. Thus, hydroxyectoine becomes more important for cells grown under high salinity and high temperature conditions. The finding that an *ectD *mutant is thermosensitive, but not osmosensitive, confirms that the hydroxyectoine synthesized by EctD is essential for thermoprotection of *C. salexigens *[33].

The observation that *C. salexigens *accumulates trehalose under high temperature conditions was unexpected, as only traces of this solute can be detected in the wild type strain grown at 37°C [22,26,35]. This compound is a general stress protectant, including heat stress, that is widely spread in both prokaryotic and eukaryotic microorganisms [15]. Within the genome of *C. salexigens*, there are orthologs of the *otsA *and *otsB *genes, encoding the enzymes for trehalose synthesis from glucose. Trehalose is also accumulated at 37°C in the ectoine-deficient mutant CHR62 [18] indicating that ectoine suppresses, either directly or indirectly, trehalose synthesis in the wild type strain (Fig. 1).

### Regulation of the synthesis of ectoines

In recent years, we have accumulated increasing evidence that ectoine(s) synthesis is a highly controlled process, integrated in the cellular regulatory network in response to (at least) osmotic and heat stress, and further regulated by extracellular solutes and iron. The elucidation of these intricate mechanisms controlling ectoine(s) synthesis, and therefore osmo- and thermoadaptation of *C. salexigens*, becomes crucial to generate modified strains improved in ectoine(s) production with prospective industrial use.

As described before, our physiological data indicate that the levels of ectoine and hydroxyectoine are maximal during the stationary phase of growth, and that ectoine accumulation is up-regulated by salinity, whereas hydroxyectoine accumulation is up-regulated by both high salt and high temperature [33]. We have now evidence that this regulation occurs, at least in part, at the transcriptional level (Fig. 1). S1 protection assays and transcriptional fusions with *lacZ *demonstrated that the ectoine synthesis genes (*ectABC*) can be expressed from two promoter regions. One is located upstream of *ectA *and composed of four putative promoters (*PectA1-4*) and the second one is an internal promoter located upstream of *ectB *(*PectB*) [35]. Besides, a transcriptional fusion between the region upstream of *ectD *and *lacZ *is expressed in *E. coli*, indicating the existence of promoter activity (M. Reina-Bueno et al. unpublished results).

Expression of *PectA-lacZ*, *PectB-lacZ *[35] and *PectD-lacZ *(Reina-Bueno et al., unpublished results) transcriptional fusions are maximal during stationary phase of growth, in agreement with our physiological data. In addition *PectA*, *PectB *[35] and *PectD *(unpublished results) show promoter activity at low salinity, suggesting that *ectABC *and *ectD *may be partially constitutive systems. *PectA *expression is osmoregulated (in an *E. coli *background), and the S1 protection assays suggest that *PectA1*, *PectA3 *and *PectA4 *may be the osmoregulated promoters within the *PectA *region. Expression of *PectA *and (especially) *PectB *is induced by continuous growth at high temperature, and repressed in the presence of osmoprotectants (betaine and ectoine), the DNA gyrase inhibitor nalidixic acid, and an excess of iron (only *PectA*) [35]. Finally, *PectD *expression is osmoregulated and thermoregulated, as suggested by real-time PCR analysis (Reina-Bueno et al., unpublished results).

The above data indicate that transcription of ectoine(s) synthesis genes involve multiple promoters which allow the system to be regulated by many environmental factors including salinity, temperature, external osmoprotectants, and iron (Fig. 1). Ensuring appropriate expression to this changing environment needs the involvement of a number of transcription factors belonging to different regulatory pathways. The *in silico *analysis of the -10 and -35 sequences of the regions upstream of *ectA*, *ectB *and *ectD *showed that *PectA1 *and *PectA2 *overlap with putative recognition sites of both the main vegetative factor σ^70 ^[35] and the iron homeostasis regulator Fur (Argandoña et al., unpublished results), whereas *PectA3 *is similar to σ^S^-dependent promoters [35], and *PectB *[35] and *PectD *(Reina-Bueno et al., unpublished results) may be recognized by the heat stress factor σ^32^. In agreement with these predictions, expression of *PectA-lacZ *and *PectD-lacZ *fusions depend partially on the general stress factor σ^S ^and the specific heat stress factor σ^32^, respectively, at least in an in *E. coli *background [35] (Reina-Bueno et al., unpublished results). Both *C. salexigens *and *E. coli *belong to the Gammaproteobacteria, and their σ^S ^and σ^32 ^proteins show a high degree of similarity, suggesting that they could play similar roles in both bacteria.

An additional element involved in the transcriptional control of ectoine hydroxylation seems to be the product of the gene *ectR*, located upstream of *ectD *(Figs 1 and 3). We have found that a *C. salexigens ectR *strain grown under high salt and temperature conditions accumulates less hydroxyectoine if compared to the wild type strain (Reina-Bueno et al., unpublished results). These data suggest that EctR is a transcriptional activator that might interact with the promoter region upstream of *ectD*.

Although temperature induction of *PectA *and *PectB *expression initially suggested that, in addition to osmoprotection, ectoine might have a physiological role in thermoprotection of *C. salexigens *[35], we later found that ectoine levels do not increase in response to temperature [33]. One explanation for this is that at high temperature ectoine is rapidly converted to hydroxyectoine by *C. salexigens *and therefore ectoine accumulation decreases in response to temperature. This is compatible with temperature induction of the ectoine synthesis genes, since ectoine is the precursor of hydroxyectoine.

In agreement with the general assumption that transport of compatible solutes is preferred over the synthesis, because the latter is energetically less favourable to the cells [36], *C. salexigens *cells growing with betaine do not accumulate ectoine(s) at any salinity tested [26,35]. As betaine only represses partially the expression of *PectA *and *PectB*, the existence of a post-transcriptional control mechanism that might operate at the level of enzyme activity is inferred (Fig. 1). However, other alternative mechanisms such as an increase efflux of ectoine in the presence of betaine cannot be ruled out.

## Conclusion

*C. salexigens *needs a fine tuning of its cytoplasmic compatible solute pool in order to cope with a number of abiotic stresses, including high salinity and supra-optimal temperatures. This is achieved by a highly hierarchical accumulation of solutes, starting with external osmoprotectants such as betaine, which totally suppress ectoine(s) synthesis, and followed by endogenous solutes, mainly ectoine and hydroxyectoine. In turn, ectoine(s) inhibit trehalose synthesis, but this is derepressed in response to heat stress. Ectoine and hydroxyectoine are essential for osmoprotection and thermoprotection, respectively. Thus, the traditional role attributed to ectoines as osmoprotectants has been now expanded to protection against other abiotic stresses such as high temperature, or their use as nutrients. In addition to salt and heat stress, ectoine synthesis is transcriptionally modulated by an excess of iron or external osmoprotectants. All these findings, while raising our interest to elucidate the mechanisms governing the synthesis of ectoines, suggest the existence of multiple signal transduction pathways, controlled by general (i.e. σ^S^, σ^32^, Fur) or specific (i.e. EctR) regulators, and predict the need of additional tools (i.e. wide expression analysis, mathematical modelling) in order to achieve a global comprehension of how the whole system is regulated.

## Competing interests

The authors declare that they have no competing interests.

## Authors' contributions

CV and JJN conceived the study, participated in its design and drafted the manuscript. MA, MRB, JRM and CFA have made substantial contributions to acquisition, analysis and interpretation of data concerning uptake of osmoprotectants (JRM), analysis of *C. salexigens *genome (MA, JRM), characterization of ectoine (CFA) and hydroxyectoine (MA, MRB) synthesis genes, and regulatory studies (MA, MRB). All authors read and approved the manuscript.
